# Anti-Inflammatory Effects of the Iron Chelator, DIBI, in Experimental Acute Lung Injury

**DOI:** 10.3390/molecules27134036

**Published:** 2022-06-23

**Authors:** Christian Lehmann, Nazli Alizadeh-Tabrizi, Stefan Hall, Sufyan Faridi, Irene Euodia, Bruce Holbein, Juan Zhou, Valerie Chappe

**Affiliations:** 1 Department of Physiology & Biophysics, Dalhousie University, Halifax, NS B3H 1X5, Canada; nazli.tabrizi@dal.ca (N.A.-T.); shall@dal.ca (S.H.); valerie.chappe@dal.ca (V.C.); 2 Department of Microbiology & Immunology, Dalhousie University, Halifax, NS B3H 1X5, Canada; sfaridi@dal.ca (S.F.); irene.euodia@dal.ca (I.E.); beholbein@sympatico.ca (B.H.); 3 Department of Anesthesia, Pain Management and Perioperative Medicine, Dalhousie University, Halifax, NS B3H 1X5, Canada; juanzj@dal.ca

**Keywords:** acute lung injury, ARDS, cytokine storm, ROS, iron chelation, microcirculation

## Abstract

Iron plays a critical role in the immune response to inflammation and infection due to its role in the catalysis of reactive oxygen species (ROS) through the Haber-Weiss and Fenton reactions. However, ROS overproduction can be harmful and damage healthy cells. Therefore, iron chelation represents an innovative pharmacological approach to limit excess ROS formation and the related pro-inflammatory mediator cascades. The present study was designed to investigate the impact of the iron chelator, DIBI, in an experimental model of LPS-induced acute lung injury (ALI). DIBI was administered intraperitoneally in the early and later stages of lung inflammation as determined by histopathological evaluation. We found that lung tissues showed significant injury, as well as increased NF-κB p65 activation and significantly elevated levels of various inflammatory mediators (LIX, CXCL2, CCL5, CXCL10, IL-1𝛽, IL-6) 4 h post ALI induction by LPS. Mice treated with DIBI (80 mg/kg) in the early stages (0 to 2 h) after LPS administration demonstrated a significant reduction of the histopathological damage score, reduced levels of NF-κB p65 activation, and reduced levels of inflammatory mediators. Intravital microscopy of the pulmonary microcirculation also showed a reduced number of adhering leukocytes and improved capillary perfusion with DIBI administration. Our findings support the conclusion that the iron chelator, DIBI, has beneficial anti-inflammatory effects in experimental ALI.

## 1. Introduction

Although the clinical picture of acute lung injury (ALI) has been well described for more than 50 years, its medical definition has undergone many changes. Based on the Berlin definition of Acute Respiratory Distress Syndrome (ARDS), ALI was most recently reclassified as moderate or mild ARDS [1]. ARDS etiology is separated in two main categories. Lung injury can be induced directly (locally), as in pneumonia, smoke inhalation, and aspiration, with mainly epithelial injury, or indirectly (systemically), induced by blood-borne insults like sepsis and pancreatitis with mainly endothelial injury [2]. ARDS results in disruption of the lung endothelial and epithelial barriers inducing increased pulmonary permeability, and impairment of pulmonary gas exchange [3]. Importantly, ARDS represents a complication of pneumonia caused by the virus SARS-CoV-2, and a significant number of patients with severe COVID-19 suffer from its consequences [4,5]. A better understanding of the basic mechanisms involved in ARDS pathogenesis could facilitate the development of potential treatments for ARDS following SARS-CoV-2 and other infectious or inflammatory conditions of the lungs.

One of the most common rodent ALI models is through the intranasal administration of endotoxin (synonym: lipopolysaccharide, LPS). Endotoxin, a pathogen-associated molecular pattern (PAMP) derived from the outer membrane of Gram-negative bacteria, is an extremely biologically active substance that contributes to the activation and release of inflammatory mediators and serves as an early warning signal of bacterial infection [6]. The molecular response to LPS is initiated after LPS binds to a specific LPS Binding Protein (LBP) and forms the LPS:LBP complex. LBP performs as a shuttle molecule to transfer LPS to CD14, which is usually attached to the cell membrane, to split LPS into monomeric molecules and in turn presents it to the TLR4–MD-2 complex facilitating binding of LPS to its main receptor, the Toll Like Receptor 4 (TLR-4), present on monocytes and macrophages [7]. LPS binding to TLR4 then activates a series of signaling cascades that also activates the NF-κB signalling pathway [8,9]. Following phosphorylation and degradation of inhibitory Iκ-B, the activated NF-κB translocates into the nucleus to promote gene upregulation and the ensuing production of various inflammatory cytokines [10].

**Figure 1:**
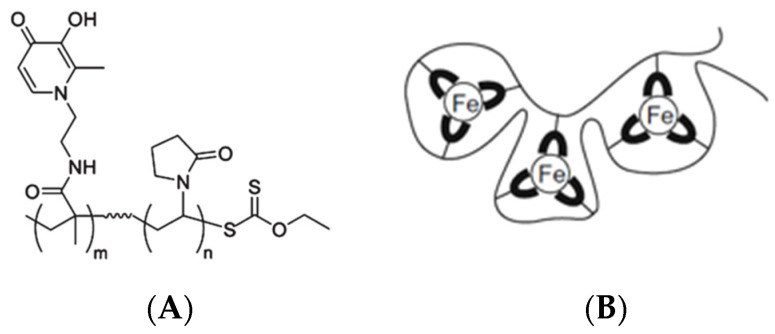
Schematic structure (**A**) of the iron chelator, DIBI (*m* = 9, *n* = 62) and its iron binding capacity (**B**).

Iron is an essential nutrient for humans and nearly all microbial pathogens. Iron also plays a critical role in the immune response to infection due to its catalytic role in the formation of reactive oxygen species (ROS), which mediate bacterial killing. However, dysregulated overproduction of ROS can be harmful and damage healthy cells. Iron chelators were initially designed to diminish the toxic effects of iron overload. More recently, iron chelators have been investigated as a potential treatment for dysregulated local and systemic inflammation through temporary reduction of iron bioavailability. DIBI is a modified hydroxypyridinone 3-hydroxy-1-(ß-methacrylamidoethyl)-2-methyl-1(1 H)-pyridinone polymer with a relatively low molecular weight (9 kDa), as shown in Figure 1A, and each molecule is capable of binding three molecules of iron (Figure 1B). DIBI is water soluble and has a low toxicity profile in comparison to conventional iron chelators. We hypothesized that DIBI attenuates pulmonary inflammation in experimental ALI via suppression of the ROS redox-sensitive NF-κB pathway. Lung tissues were studied ex vivo by histological and cytokine analyses and in vivo by intravital microscopy.

## 2. Results

### 2.1. Tissue Damage by LPS

**Figure 2:**
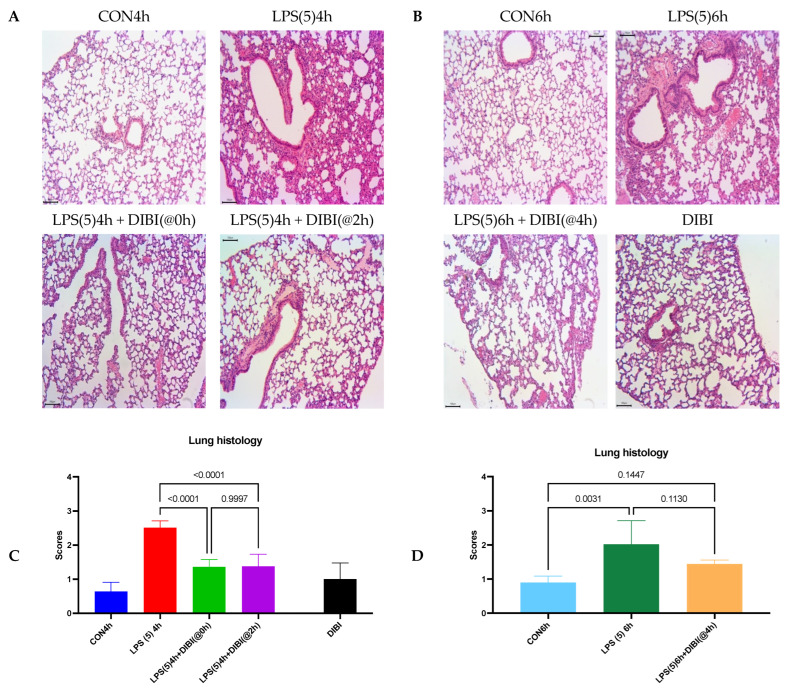
DIBI attenuated the histological lung injury induced by intranasal LPS in mice. Histopathological changes were observed using light microscopy of H&E-stained lung tissue sections 4 h (**A**) and 6 h (**B**) after LPS instillation with or without DIBI treatment as indicated. (**C**,**D**) Lung injury scores (0–4) were used to semi-quantitatively evaluate the histopathological injury determined on H&E sections. Data are expressed as means ± SD for 10 separate images per lung (*n* = 5–9 mice per group), and *p* values are indicated on top of each comparison bar. Groups: CON4h—control group observed after 4 h; LPS(5)4h—LPS 5 mg/kg observed after 4 h; LPS(5)4h + DIBI(@0h)—LPS 5 mg/kg and DIBI 80 mg/kg administration at time 0 h observed at 4 h; LPS(5)4h + DIBI(@2h)—LPS 5 mg/kg and DIBI 80 mg/kg administration at time 2 h observed at 4 h; CON6h—control group observed after 6 h; LPS(5)6h—LPS 5 mg/kg observed after 6 h group; LPS(5)6h + DIB(@4h)—LPS 5 mg/kg and DIBI 80 mg/kg administration at time 4 h observed at 6 h; DIBI—control animals 4 h after administration of DIBI at dosage of 80 mg/kg.

Histopathological changes were evaluated by H&E staining of lung tissue sections using a previously published scoring system. Intranasal administration of LPS resulted in significant lung injury represented by edema, alveolar hemorrhage, cellular infiltration, and thickening of the alveolar wall, with a 4 h post LPS score = 2.51 ± 0.20 and a 6 h post LPS score = 2.02 ± 0.69 in comparison to their respective control groups (CON4h and CON6h, Figure 2).

Intraperitoneal (i.p.) administration of DIBI significantly reduced the histological score of lung injury at the 4 h timepoint if given 0 or 2 h after LPS.

At 6 h post LPS, animals with DIBI administration 4 h after LPS challenge did not show a significant difference to healthy control animals. However, administration of DIBI, 4 h after LPS instillation also did not reach significance in comparison to untreated animals. DIBI-treated controls that had not received LPS displayed no evidence of tissue injury.

### 2.2. NF-κB Activation

**Figure 3:**
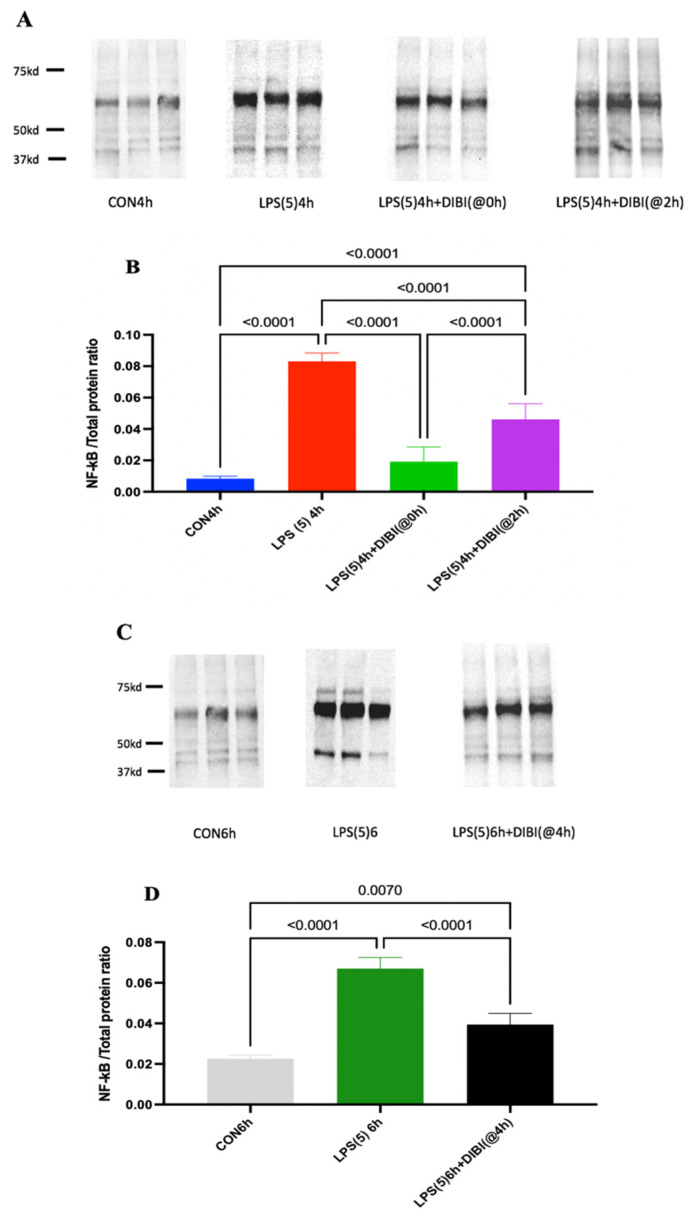
DIBI attenuated NF-kB activation in lung tissue following intranasal LPS challenge in mice. Phosphorylated NF-kB was detected using Western blotting with a specific anti-p65NFkB antibody. (**A**,**C**) Representative Western blot images. (**B**,**D**) Semi-quantification of NF-kB signal. Data are expressed as means ± SD for each group (*n* = 5–9 mice per group), *p* values for significant differences are indicated on top of each comparison bars. Groups: CON4h—control group observed after 4 h; LPS(5)4h—LPS 5 mg/kg observed after 4 h; LPS(5)4h + DIBI(@0h)—LPS 5 mg/kg and DIBI 80 mg/kg administration at time 0 h observed at 4 h; LPS(5)4h + DIBI(@2h)—LPS 5 mg/kg and DIBI 80 mg/kg administration at time 2 h observed at 4 h; CON6h—control group observed after 6 h; LPS(5)6h—LPS 5 mg/kg observed after 6 h group; LPS(5)6h + DIB(@4h)—LPS 5 mg/kg and DIBI 80 mg/kg administration at time 4 h observed at 6 h.

LPS administration significantly increased NF-κB activation in lung tissue in comparison to control at 4 and 6 h post administration (Figure 3). Early treatment with the iron chelator DIBI at 80 mg/kg, 0 or 2 h after LPS, significantly reduced NF-κB activation in lung tissue in comparison to LPS alone as observed at 4 h post LPS challenge (Figure 2, *p* < 0.0001). Late treatment with DIBI administrated at 4 h post LPS also reduced NF-κB activation significantly as evaluated at 6 h post LPS (*p* < 0.0001).

However, NF-κB levels in DIBI-treated LPS groups at both timepoints (4 and 6 h) were not reduced to the same levels as controls.

### 2.3. Cytokine Levels

There were significant increases in LIX, CXCL2, CCL5, CXCL10, and IL-6 levels in lung tissues 4 h after LPS administration relative to the control group (Table 1). Animals with early DIBI treatment at time 0 and 2 h post LPS did not show significant increases in LIX, CXCL2, CCL5, and CXCL10, IL-1β, and IL-6 levels.

Lung levels of LIX, CXCL2, CCL5, and CXCL10, IL-1β, and IL-6 were also significantly increased 6 h after LPS instillation in comparison to controls (CON6h). DIBI administration at 4 h post LPS significantly reduced the level of CXCL-2 and IL-6 in lung tissues compared to untreated LPS animals. LIX, CXCL10, and IL-1β levels were not reduced by DIBI treatment 4 h after LPS administration compared to controls.

### 2.4. Intravital Microscopy

**Figure 4:**
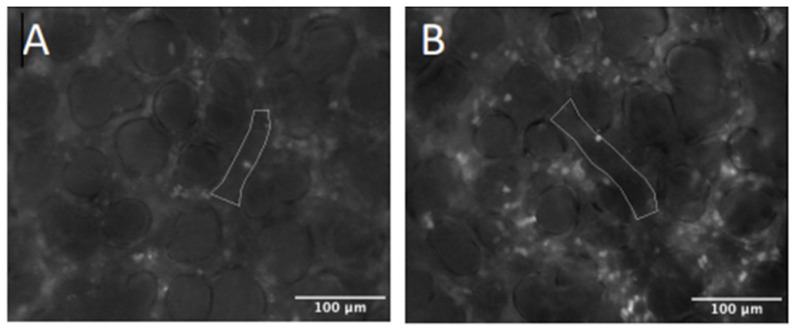
Intravital imaging of leukocyte adhesion in the pulmonary microcirculation. (**A**) Control group, (**B**) LPS group.

**Figure 6:**
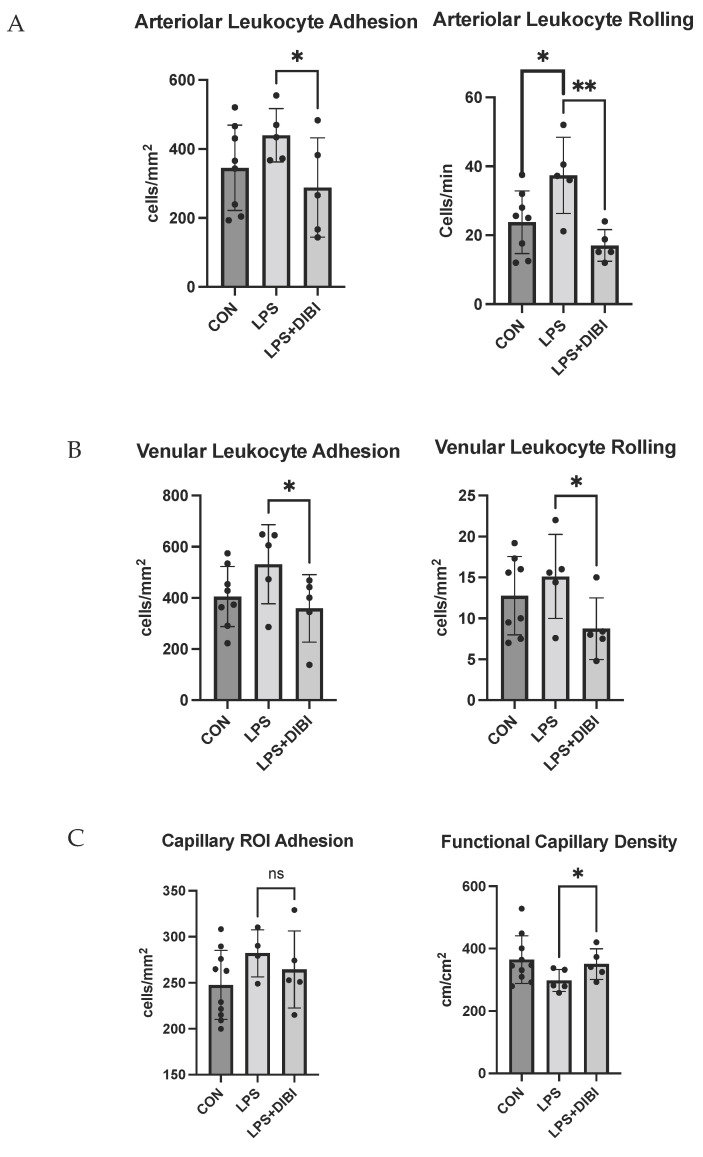
DIBI reduces leukocyte rolling and adhesion and improves capillary perfusion in the pulmonary microcirculation following intranasal LPS challenge in mice. Leukocyte–endothelial interactions (arterioles: **A**, venules: **B**, capillaries: **C** left panel) and functional capillary density (**C** right panel) were studied by lung intravital microscopy. Data are expressed as means ± SD for each group (*n* = 5–9 mice per group), significant differences are indicated on top of each comparison bars. Groups: CON—control group observed after 6 h; LPS—LPS 5 mg/kg observed after 6 h group; LPS + DIBI—LPS 5 mg/kg and DIBI 80 mg/kg administration at time 0 h observed at 6 h. * *p* > 0.05, ** *p* > 0.01.

Representative images of leukocyte adhesion and capillary perfusion in the pulmonary microcirculation are presented in Figure 4 andFigure 5, respectively. LPS administration significantly increased leukocyte rolling in lung arterioles, while DIBI treatment at 0 h resulted in a significant reduction in LPS induced arteriolar and venular leukocyte rolling and adhesion when assessed at the 6 h post LPS timepoint (Figure 6A,B).

Functional capillary density in pulmonary microcirculation was also improved following DIBI treatment in LPS challenged mice (Figure 6C).

## 3. Discussion

We demonstrated that systemic administration of the iron chelator DIBI attenuates experimental LPS-induced ALI observed by reduced histopathological lung injury, suppresses production of pro-inflammatory cytokines in lung tissue, and improves lung microcirculation. Attenuation of ALI by DIBI may be a result of suppression of NF-κB phosphorylation.

Although DIBI has been shown to have therapeutic effects in local and systemic inflammatory models [11,12,13,14,15], the molecular mechanisms involved in these effects have yet to be fully understood. Therefore, we established an experimental ALI model in mice by using intranasal endotoxin administration at a dosage of 5 mg/kg LPS and aimed to study the effects of DIBI at several treatment timepoints post LPS administration. We chose both early and later time points for the administration of DIBI, i.e., immediately post LPS administration (T0), 2 h (T2) and 4 h (T4) after LPS. The 2 and 4 h timepoints were considered to have more clinical relevance.

We observed a significant increase in NF-κB activation in lung tissues 4 h after LPS administration. DIBI treatment at early stages (0 and 2 h post LPS) significantly reduced NF-κB activation as compared to animals receiving LPS administration only. The strongest effect was observed when DIBI was administered immediately after LPS challenge (T = 0 h) with NF-*κ*B almost at control animal levels when measured 4 h post LPS administration. To evaluate the effects of DIBI treatment at later stages (4 h after LPS administration), NF-κB activation in lung tissue was compared to a separate group of LPS-treated animals after 6 h LPS exposure. Interestingly, DIBI still reduced NF-κB activation, almost to the level of the 6 h control animals. We believe that these effects are related to the ROS suppressing effects of DIBI through iron chelation since free iron promotes ROS production and it is known that NF-κB activation is ROS sensitive [16,17].

Our findings are consistent with results from other studies using different iron chelators. Lin et al. reported that treatment of cultured rat hepatic macrophages with the iron chelator, deferiprone (DFP), prevented LPS-induced NF-κB activation [18]. Another in vitro study by Aali et al. showed that treatment with high doses of DIBI (100 or 200 μM) did prevent nuclear translocation of NF-κB p65 in CF15 cells challenged with LPS. DIBI possesses a molecular size (9 kDa) which is presumably too large to normally pass through cell walls to directly lower intracellular free iron concentrations. However, by binding to extracellular iron in the immediate environment of cells, DIBI reduces overall iron bioavailability and thus, indirectly reduces labile intracellular iron concentration and ROS generation and therefore, in turn, suppresses NF-κB activation [19]. In addition, Li et al., in an experimental murine model of local inflammation, showed that yet a different chelator, deferoxamine (DFO), blocked LPS-induced nuclear translocation of the p65 subunit of NF-κB, since DFO inhibited LPS-induced NADPH oxidase, which mediated oxidative stress through an increase in levels of the catalytic NADPH oxidase subunit, p22^phox^ [20]. In a different study, Messa et al. evaluated the effect of iron chelators on NF-κB activation in myelodysplastic cells and in leukemia cell lines (K562 and HL60), these cells being characterized by a high basal NF-κB activity. They reported that NF-κB inhibition by DFO in these cell lines was not observed with DFP [21].

In the current study, we found that i.p. injection of DIBI at a dosage of 80 mg/kg at 0 and 2 h after LPS administration significantly attenuated histological evidence of tissue damage as induced by LPS when observed after 4 h of LPS exposure and resulted in less lung injury if administered 4 h post LPS. Our histopathological findings are in general agreement with those of other experimental models of inflammation. For instance, the small intestine was examined for mucosal lesions in experimental systemic inflammation induced by LPS. Thorburn et al. reported that administration of DIBI reduced mucosal damage compared to untreated LPS animals [11]. Kono et al. indicated that pre-treatment with i.p. deferasirox (DFX) reduced infiltration of inflammatory cells and exsudate in lung tissue caused by intratracheal administration of 5 mg/kg LPS. In addition, DFX alleviated acute lung inflammation by inhibiting neutrophil extracellular trap (NET) formation, which directly damages alveolar epithelial and endothelial cells [22].

To further assess the anti-inflammatory effects of DIBI in our ALI model, levels of inflammatory cytokines and chemokines were measured in lung tissues collected 4 and 6 h after LPS challenge. LPS administration without DIBI treatment resulted in substantially elevated levels of both lung cytokines and chemokines. Early treatment with DIBI (i.e., 0 or 2 h post LPS, respectively) significantly reduced lung tissue levels of LIX, CXCL2, CCL5, and IL-6. Later treatment with DIBI at 4 h post LPS, significantly diminished lung levels of CXCL2 and IL-6. Our results confirm that early treatment with DIBI is effective in lowering the release of the major inflammatory mediators. Furthermore, these results are in agreement with our findings of both the histopathological changes and levels of NF-κB activation in lung tissue. He et al. evaluated the effect of DFO on ovalbumin-induced lung inflammation exacerbated by LPS. They found that DFO did not suppress neutrophil-related responses of IL-1β, IL-6, TNF-α, KC and RANTES in broncho-alveolar lavage fluid (BALF) [23]. We did not prepare BALF samples for a stricter comparison of results. Aali et al. reported that higher concentrations of DIBI reduced the apical secretion of IL-6 in LPS-stimulated CF15 cells [19]. Furthermore, similar findings were reported in an in vitro study where higher concentrations of DFO inhibited the release of IL-1β from LPS-stimulated alveolar macrophages from both smokers and non-smokers [24].

Intravital microscopy of various organs and tissues has been performed previously to study the effects of DIBI on tissue microcirculation regarding leukocyte activation and capillary perfusion in response to LPS and other agents. Islam et al. observed a significant reduction in intestinal leukocyte recruitment and an improvement of capillary perfusion after DIBI administration during polymicrobial sepsis [25]. Lehmann et al. also reported similar findings in a sterile endotoxemia model [26]. It is known that proinflammatory signals induce ROS formation which facilitates expression of adhesion molecules, the latter triggering leukocyte rolling and subsequent adhesion [27]. Hence, reduction in leukocyte recruitment might be due to the antioxidant effect of DIBI chelating free iron and suppressing iron-promoted ROS formation [14]. LPS-induced reduction of capillary perfusion (FCD) is not only caused directly by leukocyte adhesion to endothelial cells and subsequent obstruction of microvascular blood flow, but also indirectly, e.g., due to increase of the permeability in the microvasculature (endothelial damage) resulting in the formation of edema and compression of capillary blood flow from the outside of the microvessels [28].

Some limitations can be identified in our study. First, we only tested one dose of DIBI (80 mg/kg) based on what was used in previous studies [11]. Future experiments could test different doses of DIBI to potentially improve its efficacy as well as examining more frequent administration of DIBI, i.e., to possibly compensate for its expected short half-life. Additionally, the administration route of DIBI in our study was through i.p. injection, while the LPS was administered intranasally. Inhalation of aerosolized DIBI could potentially be a more effective route of administration for this lung model. Another limitation of our findings is that experiments were performed on healthy mature adult male mice aged 12–14-week-old [29,30]. Although it has been shown that male mice demonstrated significantly greater airway responsiveness to LPS relative to females [31], it would be helpful to investigate the inflammatory lung changes in female mice. In addition, mature adult mice show cumulative immune responses following LPS exposure compared to young mice [32]. Therefore, the factors of age and sex should be regarded in the interpretation of the results.

Recent studies have reported the possible therapeutic effects of iron chelators in patients with COVID-19 infection through preventing excessive inflammatory responses and tissue damage by blocking free iron and inhibiting the oxygen radical formation and lipid peroxidation [33,34]. The results of our study also suggest the potential therapeutic use of DIBI in systemic inflammation as caused by SARS-CoV-2 through targeting the release of inflammatory mediators such as ROS and the suppression of the cytokine storm.

## 4. Materials and Methods

### 4.1. Animals

Male C57BL/6 mice were purchased from Charles River Laboratories International Inc. (Saint-Constant, QC, Canada). All mice were wild type, 12–14 weeks old, 20–30 g body weight, and were housed in ventilated plastic cage racks in a pathogen free room of the Carleton Animal Care Facility (CACF), Faculty of Medicine, Dalhousie University, Halifax, NS, Canada. Animals were kept on a 12-h light/dark cycle at 21 °C and were acclimatized for one week, prior to the experiments. All experimental procedures were approved by the University Committee on Laboratory Animals at Dalhousie University under protocol number #19-010 and were performed following the guidelines and standards of the Canadian Council on Animal Care.

### 4.2. Experimental Model

Animals were weighed prior to anesthesia. Induction of anesthesia was accomplished by i.p. injection of sodium pentobarbital (90 mg/kg, dilution: 27 mg/mL in normal saline, Ceva Sainte Animale, Montreal, QC, Canada) using a 25-G needle (BD PrecisionGlideTM). After injection, the mouse was returned to its cage on a heating pad until fully anesthetized. The pedal withdrawal reflex (response to toe pinch) was used every 15 min throughout the procedure to assess the depth of anesthesia. As needed, additional doses of sodium pentobarbital (9 mg/kg; 5.4 mg/mL) were administered.

LPS from *Pseudomonas aeruginosa* (Sigma-Aldrich, Oakville, ON, Canada, L8643, source #12180104, purified by gel-filtration chromatography) was diluted in sterile normal saline (10 mg/mL stock) and stored at 4 °C. LPS was administered at a dose of 5 mg/kg. Anesthetized mice were placed in the palm of the handler’s hand and using a pipette tip, the tongue was gently moved out and pinned down with the thumb. Small droplets of the LPS or saline solution were slowly added into the left nostril of the mouse with a pipette tip until the full volume had been instilled. After LPS instillation, the animal was placed back into the cage on the heating pad and monitored for the duration of the experiment.

#### Experimental Groups

**Figure 7:**
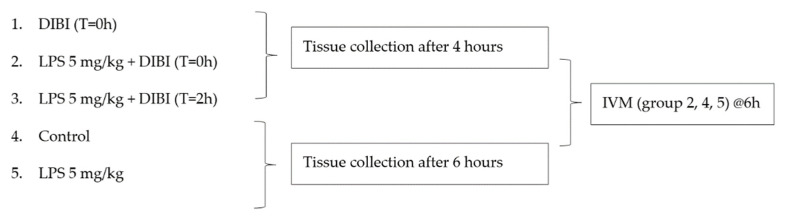
Experimental groups.

Treatment groups (*n* = 5–9 mice/group, see Figure 7) received 80 mg/kg DIBI i.p. at 0, 2, or 4 h. DIBI (Denying Iron from Bacterial Infection, as supplied by Fe Pharmaceuticals (Canada) Inc. formerly Chelation Partners Inc., Halifax, NS, Canada) is a 9 kDa MW, highly selective synthetic iron chelator, with a poly-vinylpyrrolidone backbone and containing nine 3-hydroxy-1-(β-methacrylamidoethyl)-2-methyl-4(1H) pyridinone (MAHMP) residues. One molecule of DIBI binds to 3 molecules of iron (Fe^3+^) in a fully stable hexadentate configuration. Control sham-treated animals received normal sterile saline.

### 4.3. Histology

Right lungs were fixed in 10% phosphate-buffered formalin for 3 days. Samples were then cleaned to remove all connective and muscle tissues and stored in 70% ethanol prior to embedding in paraffin blocs. Embedded tissues were sliced into 5 μm sections and mounts were dried for at least two days in an oven (56–76 °C) and then stored at room temperature before Hematoxylin and Eosin (H&E) staining. Finally, stained tissue sections were examined by light microscopy (Olympus, BH-2) and images were collected from all areas of the stained tissues. The degree of inflammation was then assessed and scored based on the presence of edema, hemorrhage, immune cells infiltration, cell wall thickening and the presence of aggregates. Scoring scale: 0, minimum damage; 1, mild damage; 2, moderate damage; 3, severe damage; and 4, intense damage [3].

### 4.4. Western Blotting

Lung samples were homogenized in cell lysis buffer of radioimmunoprecipitation assay (RIPA) buffer supplemented with protease and phosphatase inhibitor cocktail using a homogenizer (985370) on ice. The Bradford protein assay (Bio-Rad) was used to determine protein concentration in lung tissue lysates and equal amounts of protein (30 μg) were loaded, separated by sodium dodecyl sulphate polyacrylamide gel electrophoresis on 10% pre-cast gels, and transferred onto nitrocellulose membranes 0.45 μm membranes 0.45 μm (Bio-Rad, cat#1620115). Following transfer, the membranes were blocked with 5% fat-free milk for 1 h at room temperature and then incubated with specific primary antibody (1:500 NF-kB p65 (F-6): sc-8008 conjugated with HRP, Lot#B0520) allowing binding overnight at 4 °C with gentle shaking. This antibody detects both phosphorylated and non-phosphorylated P65. After washing three times with T-TBS, and then time with TBS, the protein of interest was detected by chemiluminescence with a scanner (Chemidoc, Bio-Rad, Hercules, CA, USA). The intensity of each band was analyzed using ImageJ (NIH, Bethesda, MD, USA). Normalization of the protein of interest was performed using total protein in each sample by densitometry of scanned membrane stained with Amido black.

### 4.5. Lung Tissue Inflammatory Mediators

Lung levels of selected inflammatory mediators (CCL5, CXCL2, LIX, IL-6, IL-1β, CXCL10) were analyzed using a custom-made mouse magnetic bead-based multiplex assay obtained from R&D (L#139804). Samples were run in duplicate, and the sample dilution was 1:5 (40 μL of tissue lysates mixed with 160 μL of diluent) using the Bio-Plex sample diluent. Bio-Plex instruments and Bio-Plex software (Bio-Rad, Mississauga, ON, Canada) were used according to protocols provided by the manufacturer. Finally, the plate was read using the Bio-Rad 200 luminometer with Bio-Plex manager software. Analysis with the Bio-Rad machine uses two lasers: one to excite the dyes inside each magnetic bead and identify the bead region; another one to excite the Phycoerythrin (PE) to measure the amount of analyte bound to the beads. A 488 nm laser light was used to excite PE to induce a maximum light emission of 575 nm.

Protein extraction by homogenization with 10 mL T-PER buffer and BCA assay was performed prior to the multiplex experiment to measure the amount of protein inside the tissue in each tissue sample. Additionally, normalization of each inflammatory mediators was performed after multiplex assay using the total protein content of each tissue sample.

### 4.6. IVM

The pulmonary microcirculation was imaged in vivo at 6 h post-induction. Anesthesia was provided for the duration of the procedure via isoflurane (1–5%; Fresenius Kabi, Bad Homburg, Germany). Following orotracheal intubation, animals were oriented in a right lateral decubitus position and positive-pressure mechanical ventilation was applied with a target pressure of 20 cm H_2_O and positive-end expiratory pressure of 5 cm H_2_O. Body temperature was maintained at 37.0 ± 0.5 °C and a paw pulse oximeter was applied to provide real-time assessment of blood oxygenation and heart rate. A homogeneous mixture of Rhodamine-6G (0.75 mg/kg; Sigma-Aldrich, St. Louis, MO, USA) and bovine FITC-albumin (50 mg/kg; Sigma-Aldrich, St. Louis, MO, USA) in saline was injected intravenously to visualize leukocytes and blood flow, respectively. A thoracotomy was performed to expose the left lung, at which point a vacuum-stabilized imaging window (Luxidea, Calgary, AB, Canada) was applied to stabilize the lung for imaging. Imaging was performed using a widefield fluorescent microscope (Leica, Wetzlar, Germany) fitted with 530–550 nm and 460–490 nm bandpass excitation filters. Five fields of view each of the pulmonary venules, arterioles, and capillary regions of interest (ROI) were recorded for 30 s using a black/white CCD camera. All analyses were performed in a blinded fashion as follows: leukocyte adhesion was quantified in venules, arterioles, and capillary ROI, while leukocyte rolling was quantified only in venules and arterioles. Functional capillary density (FCD) was quantified in capillary ROI.

### 4.7. Statistical Analysis

All data were analyzed using the software Prism 9 (GraphPad Software, La Jolla, CA, USA). To confirm normal distribution of data, the Kolmogorov–Smirnov Test was used. Pairwise comparisons were performed using Student’s *t*-test. One-way ANOVA followed by Tukey’s multiple comparison test was used to analyze normally distributed data for 3 or more groups. Data was expressed as mean ± standard deviation (SD). Significance was assumed at *p* values less than 0.05 (*p* < 0.05).

## 5. Conclusions

The present study investigated the anti-inflammatory effects of the iron chelator, DIBI, in an experimental model of ALI in mice. We found that DIBI administration reduced LPS-induced NF-κB activation, attenuated lung histological injury, diminished inflammatory mediator release and improved microcirculation in lung tissues. These results suggest that DIBI has potential as anti-inflammatory treatment for clinical conditions associated with acute lung inflammation including ARDS.

## Figures and Tables

**Figure 5:**
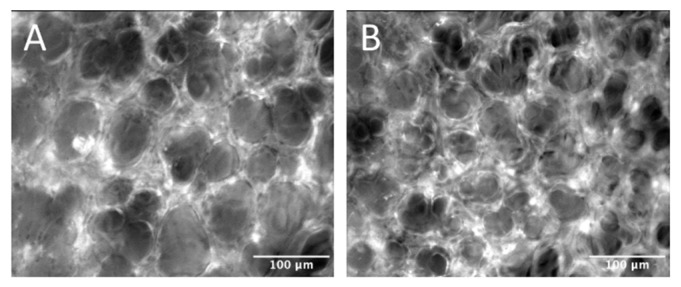
Intravital imaging of capillary perfusion in the pulmonary microcirculation. (**A**) Control group, (**B**) LPS group.

**Table 1:** Lung cytokine levels (normalized to total protein content, pg/mL, mean (SD), * *p* < 0.05 vs. CON4/6h, ** *p* < 0.05 vs. LPS(5)4/6h).

	CON4h	LPS(5)4h	LPS(5)4h + DIBI(@0h)	LPS(5)4h + DIBI(@2h)	CON6h	LPS(5)6h	LPS(5)6h + DIBI(@4h)
LIX	3.3 (2.7)	43.4 (19.2) *	21.5 (7.1) **	14.9 (4.7) **	2.1 (0.8)	25.7 (10.2) *	23.1 (8.7) *
CXCL2	0.6 (0.8)	26.1 (16.6) *	4.5 (1.4) **	9.1 (1.6) **	0.6 (0.4)	6.1 (0.6) *	3.4 (0.9) *^,^**
CCL5	3.2 (1.9)	35.6 (23.2) *	9.4 (1.1)	11.2 (2.4)	2.6 (1.6)	26.4 (11.9) *	14.1 (7.8)
CXCL10	4.9 (7.5)	38.3 (28.1) *	14.9 (5.7)	24.0 (4.6)	0.9 (0.2)	18.9 (13.2) *	22.5 (5.6) *
IL-1*β*	3.6 (0.4)	12.9 (9.2)	8.7 (1.6)	8.2 (1.8)	4.0 (1.6)	10.3 (2.7) *	8.1 (0.7) *
IL-6	1.4 (0.2)	27.9 (13.7) *	0.8 (0.4) **	1.6 (0.8) **	0.5 (0.2)	8.1 (1.1) *	2.4 (2.6) **

## Data Availability

Not applicable.

## Sample Availability

Samples of DIBI are available from the authors.
